# Gastrointestinal tolerance of low FODMAP oral nutrition supplements in healthy human subjects: a randomized controlled trial

**DOI:** 10.1186/s12937-017-0256-3

**Published:** 2017-05-25

**Authors:** Jennifer Erickson, Renee Korczak, Qi Wang, Joanne Slavin

**Affiliations:** 1 Department of Food Science and Nutrition, University of Minnesota, 1334 Eckles Ave, St Paul, MN 55108 USA; 2 Clinical and Translational Science Institute, University of Minnesota, 717 Delaware St SE, Minneapolis, MN 55414 USA

**Keywords:** Gastrointestinal tolerance, Oral nutrition supplement, FODMAP, Breath hydrogen

## Abstract

**Background:**

There has been increasing interest in utilizing a diet low in fermentable oligosaccharides, disaccharides, monosaccharides and polyols (FODMAPs) for the treatment of irritable bowel syndrome (IBS), a functional gastrointestinal disease. While studies have indicated that this diet can be effective at symptom reduction, it is a restrictive diet and patients may find it challenging to find low FODMAP products to meet their nutrient needs. The primary objective of this study was to assess the gastrointestinal (GI) tolerance of three low FODMAP oral nutrition supplements (ONS) in healthy adults.

**Methods:**

A double-blind randomized controlled crossover study was conducted in 21 healthy adults (19–32 years). Fasted subjects consumed one of four treatments at each visit, with a one week wash out period between visits. Each participant received all treatments. Treatments included three low FODMAP ONS formulas (A, B, and C) as well as a positive control consisting of 5 g fructooligosaccharides (FOS) mixed in lactose-free milk. Breath hydrogen was measured at baseline, 1, 2, 3, and 4 h post treatment consumption. Subjective GI symptom questionnaires were completed at baseline, 0.5, 1, 1.5, 2, 3, 4, 12, 24 and 48 h following treatment consumption. Mean breath hydrogen concentrations and baseline corrected area under the curve for both breath hydrogen and GI symptoms were analyzed and compared between treatments. Significance was determined at *P* < 0.05.

**Results:**

The positive control resulted in higher breath hydrogen response compared to all three of the low FODMAP ONS beverages at 3 and 4 h after consumption. There were no differences in GI symptom response between treatments.

**Conclusions:**

All treatments were well tolerated in healthy participants. The low FODMAP formulas resulted in a lower breath hydrogen response compared to the positive control, and may be better tolerated in individuals with IBS. More research should be conducted to better understand the GI tolerance of low FODMAP ONS in individuals with IBS.

**Trial registration:**

The protocol for this study was registered on ClinicalTrials.gov in January 2016 (Clinical Trials ID: NCT02667184).

## Background

Irritable bowel syndrome (IBS) is a prevalent functional gastrointestinal (GI) disorder impacting 11.2% of individuals worldwide [1]. The disease presents as various GI symptoms including abdominal pain and changes in stool consistency and frequency [2]. While many medications and therapies exist to treat the symptoms of IBS, no cure currently exists [3]. Recently, clinical research has focused on diet as a treatment for IBS, since food can be related to symptom expression in many patients [4]. Diets low in fermentable oligosaccharides, disaccharides, monosaccharides and polyols (FODMAPs) are recommended treatment options in Australia and the United Kingdom to manage the symptoms of IBS [5, 6]. FODMAPs are not readily absorbed in the small intestine causing fluid to be pulled into the intestinal lumen, and the remaining carbohydrates to be fermented in the colon causing gas production [7]. By removing the carbohydrates with these properties from the diet, patients often see a reduction in symptoms. Halmos et al. reported clinically significant symptom improvement in 70% of participants with IBS while following a low FODMAP diet [8].

The main advantage to following a low FODMAP diet is that it can greatly improve symptoms; however, the diet can also be very restrictive. There are not many ready-to-eat options for consumers, and almost all meals and snacks must be prepared at home. Low FODMAP diets should be initiated with the guidance of a registered dietitian (RD), to ensure the patient has the knowledge and skills to create a nutritionally complete diet [7]. Without such guidance, restrictive diets, like the low FODMAP diet, may leave consumers focused on only a few foods that they know are well tolerated. Limited diets may result in nutrient deficiencies [9, 10]. While well tolerated, the low FODMAP diet is complex and the broad availability of convenient food solutions are limited today. Oral nutrition supplements (ONS) are liquid beverages formulated to improve the nutrient consumption of those individuals with either minor nutritional gaps or specific disease conditions. While several types of ONS already exist on the market, it is very difficult to find an ONS that is low in FODMAPS. Low FODMAP ONS may grow in demand, providing a good source of nutrition and serve as a convenient and healthy alternative to solid food for individuals who suffer from IBS and struggle to meet their nutritional needs with conventional foods.

While previous studies have assessed the effects of enteral nutrition formulas with varying FODMAP contents [11–13], no prior studies have examined the acute gastrointestinal tolerance of ONS beverages that have been formulated to be low in FODMAPs. Therefore, in this pilot study, our aim was to examine the gastrointestinal tolerance of three low FODMAP formulated ONS (A, B and C) in 21 healthy human subjects. We compared the three low FODMAP ONS to an isocaloric, positive control consisting of lactose-free milk mixed with 5 g of fructooligosaccarides. We hypothesized that the consumption of the low FODMAP ONS would produce a lower breath hydrogen response compared to the positive control. Additionally, we hypothesized that the subjective reports of gastrointestinal symptoms would be lower following the consumption of the low FODMAP supplements compared to the positive control.

## Methods

### Study design

The study was reviewed and approved by the University of Minnesota Institutional Review Board, Human Subjects Committee. The protocol for this study was registered on ClinicalTrials.gov in January 2016 (Clinical Trials ID: NCT02667184). The study design was a randomized, controlled, crossover study with 21 subjects (11 males, 10 females). The study consisted of four visits, assessing the effects of three low FODMAP ONS and one positive control, with a seven day washout period between each visit. Participants received each of the four treatments only once. Treatments were randomized, coded and blinded to both participants and researchers. Treatment codes were not revealed until following the statistical analysis.

### Subjects

Subjects were recruited via flyers displayed around the University of Minnesota campus in Saint Paul and Minneapolis. Prior to enrollment, interested individuals were screened to determine if he or she met all of the eligibility criteria. Eligible participants were between the ages of 18 and 65 with a BMI between 18.5 and 29 kg/m^2^ with the ability to provide written, informed consent after review of study protocol and procedures. Exclusion criteria included the use of enemas, laxatives, proton pump inhibitors, or antibiotics within the past 3 months, history of past or current gastrointestinal conditions, high fiber consumption, use of tobacco products and regularly skipping breakfast and/or lunch. Applicants with recent weight fluctuations of more than 10 pounds, known allergies to any ingredients in the treatments, or recent participation in another dietary intervention trial were excluded. Subjects meeting all of the inclusion and exclusion criteria were enrolled in the study. Informed consent was obtained from each participant before the commencement of the study.

### Treatments

We tested three different ONS beverages that were all formulated to be low in FODMAP concentration, formulas A, B and C. Each of the low FODMAP formulas contains less than 0.5 g FODMAPS per serving (8 oz). The positive control beverage was 8 oz of lactose-free whole milk with 5 g of fructooligosaccharides (FOS) and 2.7 g of sucrose added to match for calories. Serving size was determined based on the typical serving size of ONS beverages. FOS is a prebiotic fiber that is commonly added to enteral formulas for putative GI benefits [14, 15]. The positive control contained a known FODMAP dose of 5 g, exceeding the recommended daily limit of FODMAPs (3 g) (Table 1).

**Table 1:** Nutrient compositions of the treatment beverages

Formula	Calories	Carbohydrates (g)	Fiber (g)	Protein (g)	Fat (g)
Low FODMAP A	170	19	3	15	4
Low FODMAP B	180	22	3	15	4
Low FODMAP C	170	19	3	15	4
Lactose-free Milk + 5 g FOS and 2.7 g sucrose	170	16	5	8	8

The low FODMAP supplements contain 3 g of fiber, sourced from partially hydrolyzed guar gum and gum acacia. These fibers are slowly fermented and have been shown to be well tolerated in clinical studies [16, 17]. Daily consumption of partially hydrolyzed guar gum has been shown to improve GI symptoms in IBS patients [18].

### Hydrogen breath tests

Carbohydrate that is not absorbed by the GI tract is fermented by bacteria in the GI tract. The fermentation process results in hydrogen production as a byproduct, which is then absorbed by the intestine, transferred through the blood to the lungs where it is expired. Approximately15-30% of the population contains *Methanobrevibacter smithii*, a microorganism that converts hydrogen to methane, which would then be absorbed and expired [19]. Hydrogen breath tests measure hydrogen and methane expired from the lungs to quantify the amount of fermentation occurring in the gut [20]. A breath hydrogen increase of 20 ppm is generally indicative of symptom induction [21].

Participants were instructed to breathe into breath collection bag, and 20 ml of the end expiratory air was removed and tested. The samples were analyzed using the Quintron GaSampler System (Quintron Instruments, Milwaukee, WI). Samples were analyzed for hydrogen and methane content in duplicate and averaged to improve accuracy.

### Gastrointestinal symptom questionnaires

GI tolerance of the four beverages was established through the continual completion of GI symptom questionnaires. Bonnema et al. observed reported GI symptoms in healthy participants over a two day period following an oligosaccharide treatment [22]. For this reason, we instructed participants to complete GI symptom questionnaires for 48 h after treatment consumption. We used a modified version of the GI symptom questionnaire validated by Bocenschen et al. [23]. Participants were asked to evaluate the perceived intensity or frequency of their symptoms. Symptoms measured included gas or bloating, nausea, flatulence, diarrhea or loose stools, constipation, gastrointestinal rumbling and gastrointestinal cramping. Participants could report each symptom as none, mild, moderate, quite a lot, severe, very severe, or unbearable. Symptom scores for each time period were added to create a composite GI symptom score.

### Study procedures

Prior to the first visit, enrolled subjects received instructions to follow a low-fiber diet and avoid sugar alcohols and other sources of FODMAPs such as apples, pears, etc. for 24 h before each visit. Participants were also asked to avoid excessive exercise during the 24 h prior to each test visit. Participants were instructed to begin fasting at 7:00 pm the night before the test visit, not eating or drinking anything other than water before arriving to the testing facility.

Treatments were blinded to both investigators and subjects. Treatment order was randomly assigned by the research statistician. Treatments were portioned into opaque cups with lids and straws by a researcher with no other role in the study to conceal any visual differences between treatments from researchers and participants.

Upon arrival to the research facility, subjects completed their first breath test and GI questionnaire at baseline, prior to treatment consumption. Subjects were then given their treatment beverage and were instructed to consume the entire portion within 10 min. Additional subjective GI questionnaires were completed at the following time points: 30, 60, 120, 180, and 240 min, as well as at 12, 24, and 48 h after consumption of the test beverage. Breath hydrogen was measured at 60, 120, 180, and 240 min after ingestion of the treatments. Participants were able to return to leave the testing facility and continue with their normal daily routine after the completion of their 240 min breath hydrogen measurement. Participants were scheduled for three return visits no sooner than one week apart. The same procedures were repeated at each visit.

### Statistical analysis

Subjective GI symptoms and breath hydrogen were expressed as a change from baseline and will be compared using a baseline corrected area under the curve (AUC). Breath hydrogen measures were also compared at each individual time point. Repeated measures analysis of variance (ANOVA) was performed to evaluate whether the means were significantly different among the four treatments. If the overall F test was significant, pairwise comparisons were conducted to assess which means differ from which other means. *P-*values for pairwise comparisons were adjusted with Tukey-Kramer adjustment to account for multiple comparisons. All analysis was performed using Statistical Analysis Software (version 9.3, SAS Institute Inc., Cary, NC). A two-sided *p*-value < 0.05 was considered statistically significant.

## Results

### Subject demographics

Twenty-two subjects (11 males, 11 females) were recruited and enrolled in the study. One female subject was dropped from the study after the first visit due to the initiation of antibiotics. The 21 subjects who completed the study and were included in statistical analysis had an average age of 21.9 ± 3.7 years and average BMI of 23.3 ± 2.4 kg/m^2^.

### Hydrogen breath tests

**Fig. 1:**
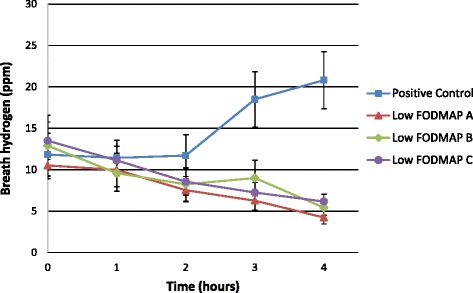
Breath hydrogen response following consumption of low FODMAP ONS beverages and positive control in healthy adults

There was no difference in baseline breath hydrogen measures between treatments (*P* = 0.86). Baseline breath hydrogen levels for all treatments were elevated, suggesting that the lead in diet insufficiently restricted fermentable carbohydrates prior to the study visits. None of the three low FODMAP ONS produced an increase in breath hydrogen production in the four hours following consumption. However, the positive control did produce an increase in breath hydrogen of 9 ppm baseline to peak (Fig. 1). The breath hydrogen AUC was statistically different between the positive control and Low FOMDAP B (10.6 vs −15.6 respectively) and the positive control and Low FODMAP C (10.6 vs −17.26) formulas after pairwise comparisons (*P* = 0.040 and 0.026 respectively). Additionally, the mean breath hydrogen level was statistically different between the positive control and each of the three low FODMAP ONS at three and four hours post consumption (Table 2).

**Table 2:** Pairwise comparisons of breath hydrogen measures at 3 and 4 h post treatment consumption

Treatment	vs. Treatment	Difference of means (3 h)	*P* value (3 h)	Difference of means (4 h)	*P* value (4 h)
Positive Control	Low FODMAP A	12.2381	0.0009^a^	16.5714	<.0001^a^
Positive Control	Low FODMAP B	9.5000	0.0143^a^	15.3810	<.0001^a^
Positive Control	Low FOMDAP C	11.2619	0.0026^a^	14.6429	<.0001^a^
Low FODMAP A	Low FODMAP B	−2.7381	0.8051	−1.1905	0.9696
Low FODMAP A	Low FOMDAP C	−0.9762	0.9885	−1.9286	0.8860
Low FODMAP B	Low FODMAP C	1.7619	0.9380	−0.7381	0.9924

^a^Indicates significance at 0.05

### Gastrointestinal symptom questionnaires

No symptoms were reported as “severe”, “very severe” or “unbearable” by any of the participants following any of the treatments. Overall, each of the treatment beverages was well tolerated by the healthy participants. There were no significant differences in AUC responses of any of the individually measured symptoms or the composite GI symptom score between treatments. Differences in AUC measures were analyzed at both the first four hours post consumption as well as 48 h post consumption (Table 3).

**Table 3:** AUC measurements of gastrointestinal symptoms following consumption of low FODMAP ONS beverages and positive control

	Positive Control	Low FODMAP A	Low FODMAP B	Low FODMAP C		
Symptom	Mean	Mean	Mean	Mean	SE	*P* value
*Baseline- 4 h post consumption*						
Gas/bloating	0.25	−0.08	0.61	−0.21	0.37	0.40
Nausea	0.24	−0.06	0.08	−0.23	0.23	0.79
Flatulence	0.24	−0.20	0.13	0.27	0.33	0.77
Diarrhea/loose stools	0.00	−0.18	0.02	0.00	0.09	0.36
Constipation	0.24	−0.18	0.02	0.05	0.09	0.29
GI rumbling	0.21	−0.12	−0.15	0.04	0.45	0.93
GI cramping	−0.08	0.07	−0.07	−0.08	0.14	0.82
Composite GI symptom score	0.61	−0.71	0.61	−0.17	0.82	0.61
*Baseline- 48 h post consumption*						
Gas/bloating	−2.42	−5.89	4.03	−5.83	3.73	0.21
Nausea	−6.07	−4.25	−0.77	−4.42	3.94	0.81
Flatulence	−1.02	−4.49	2.99	1.23	4.04	0.59
Diarrhea/loose stools	2.67	−1.13	0.50	0.95	1.79	0.52
Constipation	0.02	−1.42	1.36	0.05	1.32	0.54
GI rumbling	−4.26	−9.64	−11.20	−5.77	5.81	0.82
GI cramping	−2.18	0.07	−1.69	−1.32	2.02	0.88
Composite GI symptom score	−10.92	−26.24	−4.82	−13.40	9.91	0.48

## Discussion

The lack of a positive breath hydrogen response following the consumption of the low FODMAP ONS demonstrates that these products are not rapidly fermented in the colon. There was a significant difference in breath hydrogen concentration at 3 and 4 h post-consumption between each of the three low FODMAP formulas and the positive control. This finding was expected as the ONS were formulated with ingredients that are known to be well tolerated and easily digested, while the positive control was made with FOS a rapidly fermentable prebiotic fiber. The positive control did produce a positive breath hydrogen response over the four hour time period; however, the 9 ppm increase (baseline to peak) was not large enough to elicit a symptomatic response in the healthy participants. Bonnema et al. observed similar findings when providing fibers to healthy subjects, as the 5 g dose of FOS did not elicit significantly greater GI symptoms compared to control [22]. While a 10 g dose of FOS has been shown to produce GI symptoms in healthy individuals, a 5 g dose was a more realistic dose for a typical ONS [22].

This study was conducted in healthy human subjects as opposed to subjects suffering from IBS. While the findings of this study provide insight into the effects of gastrointestinal tolerance of these low FODMAP formulas without confounding effects from GI disorders, it does not explain the effects of the supplements in individuals with IBS. Findings published by Magge et al. suggest that healthy individuals and individuals with IBS have similar breath hydrogen responses to low and high FODMAP diets [24]. Breath hydrogen levels remained low after consuming low FODMAP foods, and rose after consuming high FODMAP foods in both groups. The increase in breath hydrogen after consuming high FODMAP products was more exaggerated in individuals with IBS [24]. Furthermore, while healthy individuals had no difference in GI symptoms, the participants with IBS did report more GI symptoms following the high FODMAP diet [24]. Based on these reported findings, we would anticipate that the low FODMAP formulas would be well tolerated in individuals with IBS, although this study should be repeated in an IBS population to confirm.

Unfortunately, the four-hour time period used to measure the breath hydrogen response of the treatments was not enough to see a definite peak for the positive control. As a result, the full effect of the positive control on breath hydrogen is still unknown. However, the distinct difference in response between the low FODMAP and high FODMAP beverages is still evident. Additionally, a water control treatment would have allowed for a comparison between the various treatments to a continued fasting state. However, this would have required a 16 h fast for participants which may have other unintended effects on gastrointestinal symptoms.

Another limitation of this study was the elevated baseline breath hydrogen levels seen throughout the study. Although subjects were asked to follow a low fiber, low polyol diet the day prior, and to fast for 12 h prior to each visit, this was not completely effective at achieving a low baseline breath hydrogen level. Future studies should consider providing a low FODMAP diet to participants on the days prior to study visits to improve upon these baseline measures. There were no significant differences in baseline measures between treatments and the AUC measurement was corrected for baseline measures, so this limitation likely had little impact on the overall findings of this study. This study did not test participants to ensure recruitment of hydrogen producing subjects. This is another limitation of this study and should be corrected in future research.

Because these beverages are low FODMAP, they could be incorporated into the diet of an individual who is following the elimination phase of the low FODMAP diet. This phase can be restrictive, and individuals may struggle to find ready-to-consume low FODMAP snacks to carry with them. ONS are also used for patients unable to meet their nutrient needs with food alone. Patients suffering from IBS, or those experiencing other digestive sensitivities, may benefit from the use of a low FODMAP ONS during times of inadequate calorie intake, or when the diet is very limited.

## Conclusion

Overall, the three low FODMAP formulas were well tolerated by healthy human subjects as evidenced by the lack of increased breath hydrogen production and the absence of subject reported GI symptoms. This study provides evidence to support the use of a low FODMAP ONS as an option for individuals following a low FODMAP diet. More research should be conducted in the future using participants with IBS to assess the tolerance of low FODMAP ONS in the population of interest.

## Abbreviations

ANOVA: Analysis of variance
AUC: Area under the curve
BMI: Body mass index
FODMAP: Fermentable oligosaccharides, disaccharides, monosaccharides and polyols
FOS: Fructooligosaccharides
GI: Gastrointestinal
IBS: Irritable bowel syndrome
ONS: Oral nutritional supplement
